# Ecology of West Nile virus across four European countries: review of weather profiles, vector population dynamics and vector control response

**DOI:** 10.1186/s13071-016-1736-6

**Published:** 2016-09-02

**Authors:** Alexandra Chaskopoulou, Gregory L’Ambert, Dusan Petric, Romeo Bellini, Marija Zgomba, Thomas A. Groen, Laurence Marrama, Dominique J. Bicout

**Affiliations:** 1 USDA-ARS, European Biological Control Laboratory, Tsimiski 43, Thessaloniki, 54623 Greece; 2 EID Mediterranee, 165 Avenue Paul Rimbaud, Montpellier, 34184 France; 3 Faculty of Agriculture, Laboratory for Medical Entomology, University of Novi Sad, Trg D. Obradovica 8, Novi Sad, 21000 Serbia; 4 Centro Agricoltura Ambiente “G. Nicoli”, Via Argini Nord 3351, Crevalcore, 40014 Italy; 5 Faculty of Geo-Information Science and Earth Observation, University of Twente, PO Box 217, Enschede, 7500 AE The Netherlands; 6 ECDC, European Centre for Disease Prevention and Control, Tomtebodavagen 11A, Stockholm, 17183 Sweden; 7 Biomathematics and Epidemiology EPSP-TIMC, VetAgro Sup, Veterinary Campus of Lyon, Marcy l’Etoile, F-69280 France; 8 Laue-Langevin Institute, Theory Group, Grenoble cedex 9, F-38042 France

**Keywords:** West Nile virus, West Nile fever, Europe, Ecology, Control, Modelling

## Abstract

West Nile virus (WNV) represents a serious burden to human and animal health because of its capacity to cause unforeseen and large epidemics. Until 2004, only lineage 1 and 3 WNV strains had been found in Europe. Lineage 2 strains were initially isolated in 2004 (Hungary) and in 2008 (Austria) and for the first time caused a major WNV epidemic in 2010 in Greece with 262 clinical human cases and 35 fatalities. Since then, WNV lineage 2 outbreaks have been reported in several European countries including Italy, Serbia and Greece. Understanding the interaction of ecological factors that affect WNV transmission is crucial for preventing or decreasing the impact of future epidemics. The synchronous co-occurrence of competent mosquito vectors, virus, bird reservoir hosts, and susceptible humans is necessary for the initiation and propagation of an epidemic. Weather is the key abiotic factor influencing the life-cycles of the mosquito vector, the virus, the reservoir hosts and the interactions between them. The purpose of this paper is to review and compare mosquito population dynamics, and weather conditions, in three ecologically different contexts (urban/semi-urban, rural/agricultural, natural) across four European countries (Italy, France, Serbia, Greece) with a history of WNV outbreaks. Local control strategies will be described as well. Improving our understanding of WNV ecology is a prerequisite step for appraising and optimizing vector control strategies in Europe with the ultimate goal to minimize the probability of WNV infection.

## Background

West Nile virus (WNV) is an arthropod-borne pathogen transmitted by mosquitoes that was first isolated in 1937 from the blood of a febrile woman in the West Nile district of Uganda [1]. It was in 1958 when WNV was detected in Europe from a patient in Albania and since then has been repeatedly detected in the continent with human and equine infections reported from many countries [2].

WNV infection represents a serious burden to human and animal health because of the capacity of the virus to cause unforeseen and large epidemics. Until 2004, only lineage 1 and 3 WNV strains had been found in Europe. Lineage 2 strains were initially isolated in 2004 (Hungary) and in 2008 (Austria) and for the first time caused a major epidemic of WNV infection in 2010 in Greece with 262 clinical human cases and 35 fatalities [3]. Since then, outbreaks involving WNV lineage 2 have been reported in several European countries including Italy, Serbia and Greece.

In nature the virus circulates in a sylvatic/rural cycle, between birds and ornithophilic mosquitoes particularly members of the genus *Culex*, and under certain environmental conditions it spills over to human settlements where it infects humans and equines causing large epidemics. Precipitation, temperature and landscape use/management are among the most important environmental parameters that influence the life-cycles of the mosquito, the virus, the amplifying and accidental hosts and the interactions between them [4]. Because of these features, outbreaks of WNV infection are highly sporadic and focal in nature exhibiting high variability in their development and incidence across different regions [5]. Studies are needed at local levels that compare different habitats and mosquito/vertebrate communities to determine how environmental parameters influence vector population and disease transmission dynamics and how mosquito control interventions may alter these dynamics.

To mitigate WNV transmission risk to humans and animals, European governments have been investing significant resources in medical and vector control interventions [6]. The majority of these efforts are reactive emergency response measures to reported human cases with unclear effect on the containment of the epidemic [3]. There is only a limited number of studies about the impact of vector control applications on the propagation of epidemics of WNV infection and most of them have been conducted in North America [7–9]. There is a need to build on our understanding of vector control practices against WNV vectors in Europe and analyze local experiences on the prevention and control of outbreaks in order to optimize the use of resources while minimizing the probability of WNV infection [10].

Vector Control Analysis (VeCA) is an ECDC-funded vector control research project aiming to increase our knowledge on WNV vector ecology and control in Europe. The project utilizes field data collected from three ecologically different study environments, urban/semi-urban, rural/agricultural and natural wetland across four European countries, Italy, France, Serbia and Greece (four case studies), that recently experienced WN outbreaks. This paper is the introduction to a series of papers generated from the VeCA project. The objective of this paper is to provide with an in-depth review of the study environments in relation to mosquito population dynamics, weather conditions and WNV transmission history. Local vector control strategies against epidemics of WNV infection will be described as well. In the follow-up research papers an advanced analysis of the data will be presented that will result in the development of empirical and mechanistic models for mosquito population dynamics.

## Review

### Italy: rural environment in the plain territories of Bologna, Modena and Reggio Emilia

West Nile virus infections (or West Nile fever cases) have been registered in Italy in 2008 (eight cases, lineage 1), 2009 (18 cases, lineage 1), 2010 (83 cases, lineage 1), 2011 (14 cases, lineages 1 and 2), 2012 (50 cases, lineages 1 and 2), 2013 (69 cases, lineages 1 and 2) and 2014 (24 cases, lineage 2). Some of these human cases have been reported in the plain territories of Bologna, Modena and Reggio Emilia provinces: three cases in 2009 (lineage 1), 14 cases in 2013 (lineage 2) and 4 cases in 2014 (lineage 2).

**Fig. 1:**
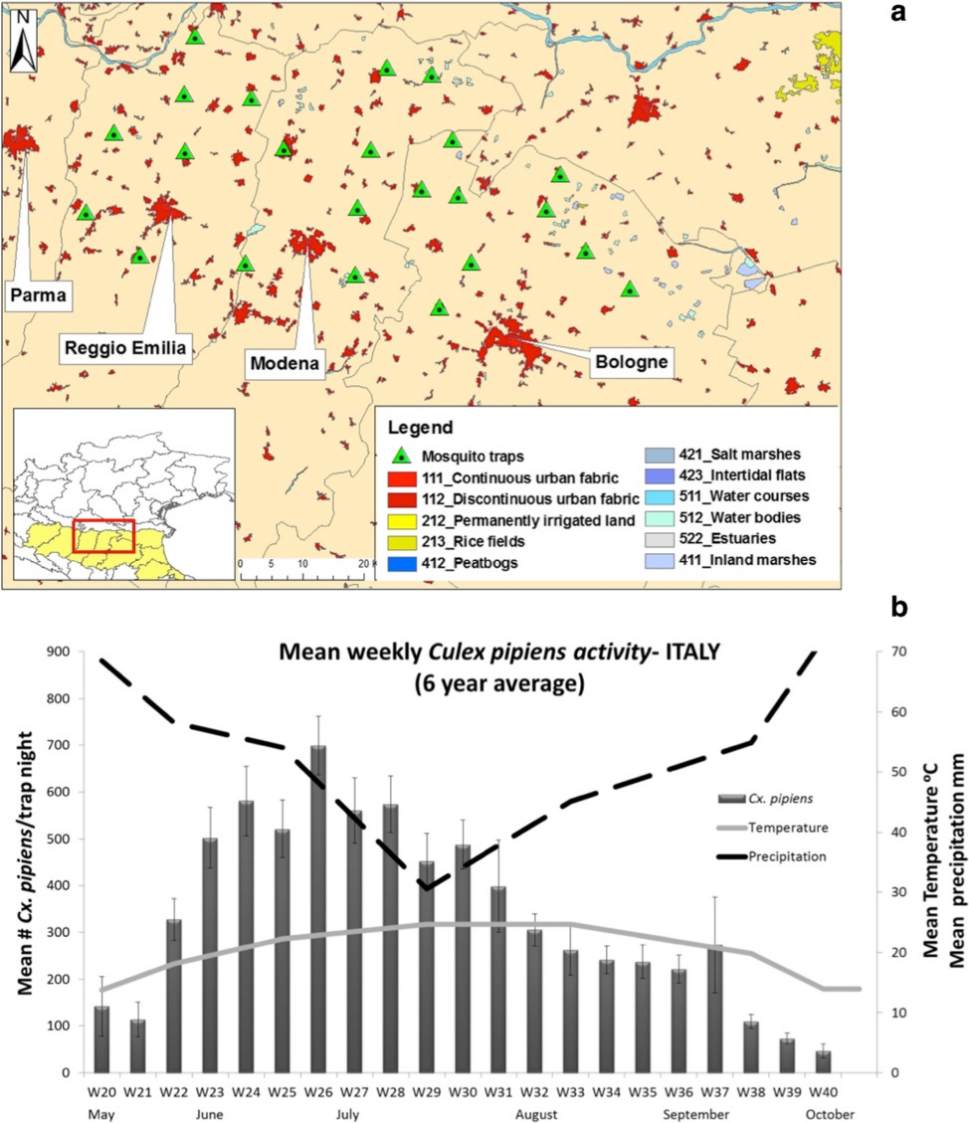
Italian West Nile virus functional unit. **a** *Culex* surveillance system with CDC traps and landscape management by CORINE. **b** *Cx. pipiens* population dynamics and weather data (6 years average)

The plain territories of Bologna, Modena and Reggio Emilia provinces are essentially rural, with a few urban localities (Fig. 1a). The study site considered in the present study covers about 500 km^2^ with a human population of about 2.2 million residents. The Po plain has a typical Mediterranean climate with rain distributed during the spring and autumn, hot dry summers and cold wet winters [11]. The most abundant mosquito species is *Cx. pipiens*, which is considered the only vector of WNV in the area [12].

The main *Culex* mosquito breeding sites are irrigation canals and ditches. Mosquito larval control operations using mostly *Bacillus thuringiensis israelensis* (*B.t.i.*) and diflubenzuron products are carried out regularly in urban and rural areas against several mosquito species including *Cx. pipiens*. Adult control applications using pyrethroid based products (i.e. deltamethrin, permethrin) are performed on a less regular basis mainly for *Aedes albopictus* control and as an emergency response following the detection of WNV in mosquitoes and birds or of WNV infection in equines and humans [10].

### France: natural wetland of southern Camargue in the Rhône Delta

Between 1962 and 1966 hundreds of cases of human and equine encephalitis due to infections of a “B Group” virus, were reported from the Rhône Delta and nearby cities [13]. A lineage 1 WNV strain was identified in 1964 for the first time in France [14] and in the late 1970’s two and five percent of the equine and human population, respectively, were positive for WNV antibodies [15]. WNV reappeared in horses in 2000 (76 cases), 2003 (five cases), 2004 (32 cases) and 2006 (five cases) [16, 17] and in humans in 2003 (seven cases) [18]. In 2015, 30 horses have shown symptoms and tested positive for WNV (our unpublished data).

**Fig. 2:**
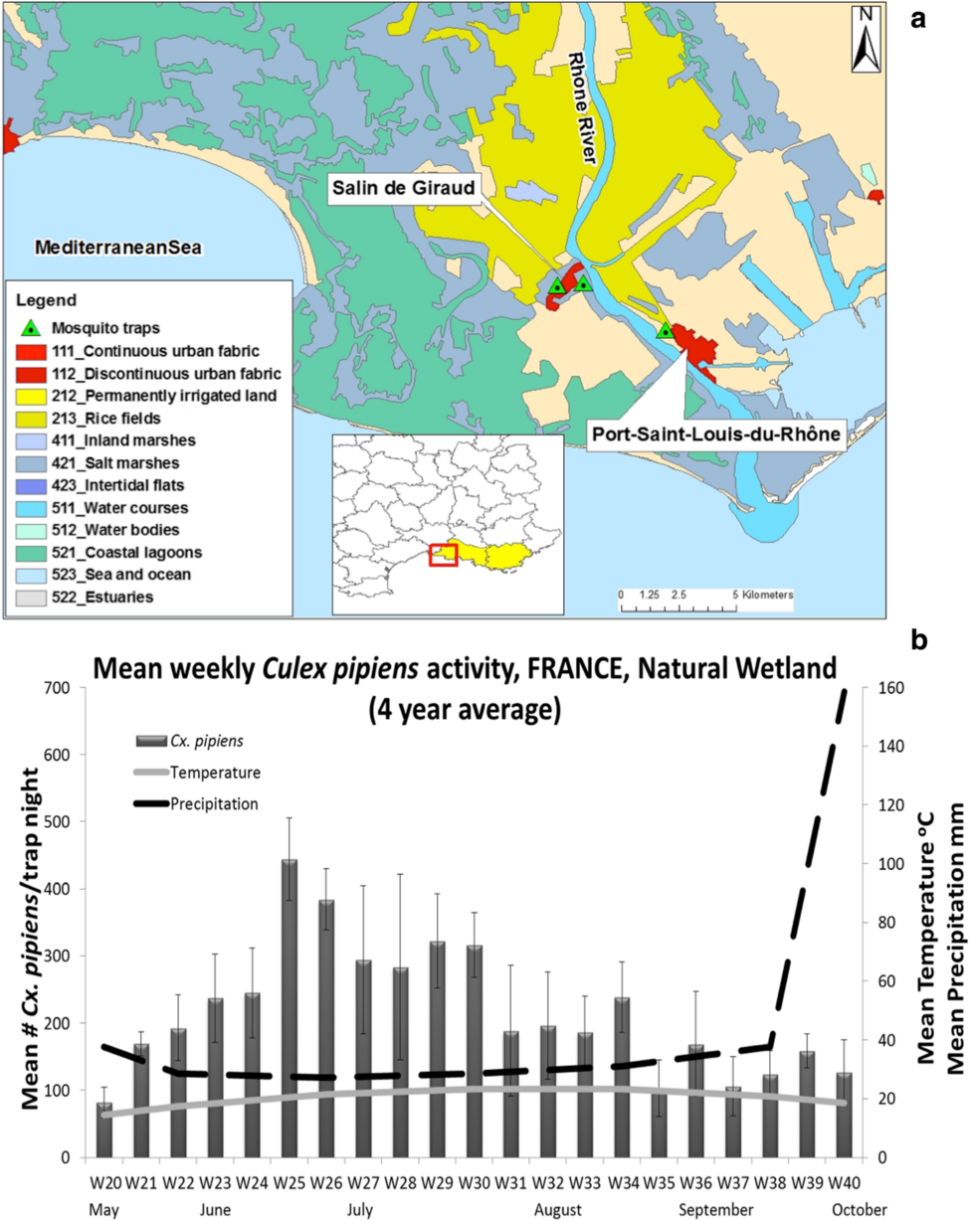
French WNV functional unit. **a** *Culex* surveillance system with CDC traps and landscape management by CORINE. **b** *Cx. pipiens* population dynamics and weather data (4 years average)

The Rhône Delta is marked by the Mediterranean climate [19]; warm and dry summers, followed by heavy autumnal rainfalls in September-October, and mild, wet winters. The study site considered in this paper, where WNV has been circulating since 2000, is located in southern Camargue, and is close to the villages of Salin-de-Giraud and Port-Saint-Louis-du-Rhône (population about 2000 and 8000, respectively) (Fig. 2a). In this part of the Rhône Delta, the most abundant and dominant mosquito breeding sites are rice fields, reed beds and flooded marshes used for hunting or bull and horse grazing/pasture. Although *Aedes caspius* is the most common mosquito throughout the delta, the species associated with WNV transmission is primarily *Cx. pipiens* followed by *Cx. modestus*. Mosquito control treatments for nuisance reduction are performed in the southern marshes of Salin-de-Giraud with *B.t.i.* products. If an outbreak of WNV infection is reported, specific treatments against the vector species are planned, on a case-by-case basis taking into consideration the entomological risk, and using either larviciding (*B.t.i.*) or adulticiding (deltamethrin) with Ultra Low Volume (ULV) applications [20].

### Serbia: urban, rural and suburban environments of Novi Sad

The first detection of antibodies against WNV in Serbia occurred in 1972, in 2.6–4.7 % of human sera [21]. The first detection of lineage 2 WNV in mosquitoes was reported from Novi Sad in 2010 [22] within the semi-urban zone of the city. The first human cases were recorded in 2012 (71 cases including nine deaths), and since then outbreaks were reported in Serbia every year in 2013 (303 cases, 35 deaths), 2014 (76 cases, 9 deaths) [23] and 2015 (5 cases, 1 death). WNV transmission was also documented in horses [24, 25] wild and sentinel birds [26].

**Fig. 3:**
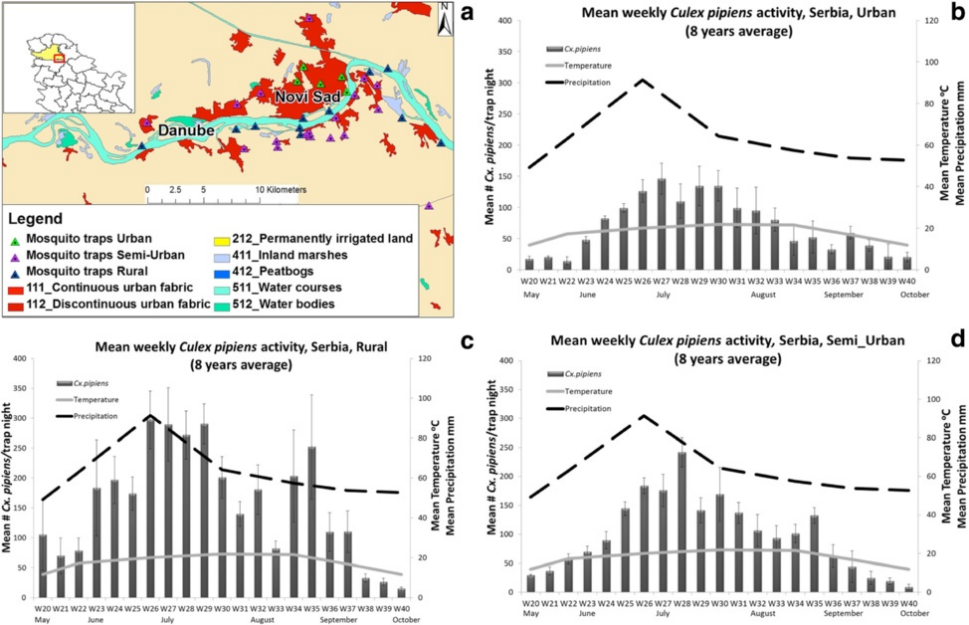
Serbian WNV functional unit. **a** *Culex* surveillance system with NS2 traps and landscape management by CORINE. **b** *Cx. pipiens* population dynamics and weather data in the urban zone (8 years average). **c** *Cx. pipiens* population dynamics and weather data in the rural zone (8 years average). **d** *Cx. pipiens* population dynamics and weather data in the semi-urban zone (8 years average)

Novi Sad is the third largest city in Serbia (population 341,625) located in the southern part of the Pannonian Plain, on the banks of the River Danube (Fig. 3a). The urban area of the city comprises 129.7 km^2^, the rest of 569.3 km^2^ is mainly agricultural land and the River Danube floodplain. Novi Sad has a temperate continental climate, with an average January temperature of -0.2 °C, a usually short and rainy spring and a summer that starts abruptly and has an average temperature in July of 21.9 °C [27].

The most common mosquito breeding sites in the rural zone around the city are channels, big puddles, old river arms and marches. Prevalent breeding sites of the semi-urban zone are drainage channels, underground sewage system, puddles, man-made containers and septic tanks. In the urban zone, the most dominant breeding sites are the underground parts of the sewage system, catch basins, flooded cellars and puddles. The most dominant *Culex* species recorded in the area is *Cx. pipiens* and is considered the primary vector of WNV.

Local vector control programs funded by the Vojvodina Province and the City Council of Novi Sad have been implemented in the region since 1974. No vector control methods targeting *Culex* spp. populations were ever implemented in the rural zone. The main *Cx. pipiens* control measures in the semi urban zone are ground larviciding and ground/aerial ULV adulticiding. In the urban zone, ground larviciding (catch basins, puddles), ground ULV adulticiding and aerial adulticiding over the urban areas and surrounding green belts are applied. From 1974 to present, temephos, pyrimiphos-methyl, diflubenzuron, *B.t.i.* and *Bacillus sphaericus* (*B.s.*) were most frequently used as larvicides and malathion, deltamethrin and lambdacyhalothrine for treatments of adult mosquitoes [6, 28].

### Greece: rural and agricultural (rice) environments of Thessaloniki

In 2010, Greece underwent for the first time an epidemic of WNV infection, the second largest in Europe in the last two decades, with 262 clinical human cases and 35 fatalities [29]. The WNV lineage 2 strain was identified from human sera, sentinel chickens, wild birds, and *Culex* mosquitoes [3, 30, 31]. The virus most likely managed to overwinter and spread fast across the country in the following years (2010–2013) resulting in more than 600 confirmed human infections and 70 deaths [32]. The agricultural region of West Thessaloniki in the Prefecture of Central Macedonia was in the epicenter of the major 2010 epidemic in Greece and up until 2013 human cases have been consistently recorded in the region. WNV transmission has also been recorded regularly (or constantly) in mosquitoes and sentinel birds (chickens, pigeons) [3, 29, 31].

**Fig. 4:**
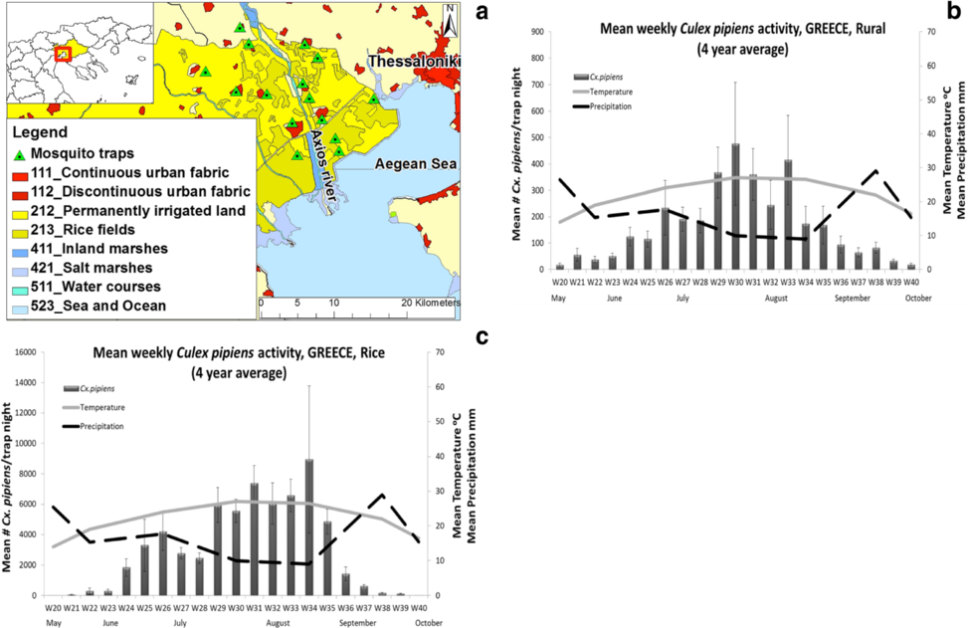
Greek WNV functional unit. **a** *Culex* surveillance system with CDC traps and land scape management by CORINE. **b** *Cx. pipiens* population dynamics and weather data in the rural/residential zone (4 years average). **c** *Cx. pipiens* population dynamics and weather data in the rice fields (4 years average)

The agricultural region of West Thessaloniki (~250 km^2^), in Northern Greece, represents a major ecosystem of Greece with rice as the dominant crop (Fig. 4a). In terms of hydrology, the territory has significant natural wetlands, rivers (Galikos, Axios, Loudias) and around 20,000 ha of rice fields (Fig. 4a). The river deltas are foci for migratory and native birds. The major mosquito breeding sites are primarily rice fields, followed by irrigation canals, and coastal marshes. The territory is adjacent to Thessaloniki, the second largest city of Greece with 1 million inhabitants. The climate of the region is mostly humid temperate with cold, rainy winters and hot, humid summers, with rare rain events [33]. *Culex pipiens* is the most dominant *Culex* species recorded in the area and most likely involved (or responsible for) in the enzootic and epidemic transmission of WNV, according to prevailing scientific evidence [3, 29, 31].

Local vector control programs, funded by the Greek government, have been implemented in the region since 1997 [6]. The main vector control methods targeting *Culex* spp. populations are aerial larviciding of the rice-fields and natural wetlands using biological and biorational products (*B.t.i.*, diflubenzuron), ground larviciding of irrigating canals, aerial, and ground ULV adulticiding using pyrethroids (deltamethrin, d-phenothrin) [29]. Adulticides applications are conducted mostly as an emergency response to WNV infections in humans.

### Mosquito and weather surveillance

**Fig. 5:**
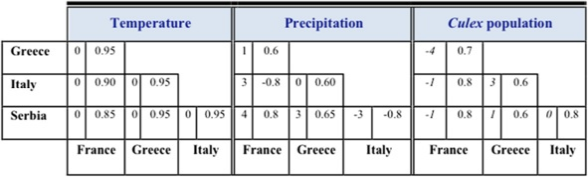
Summary of the lagged cross-correlation analysis between study sites for temperature, precipitation, and *Culex* spp. population series. Numbers at the intersection between two countries corresponds to the highest Pearson cross-correlation value (2nd column) with the associated lag period (1st column). The lag units are months for temperature and precipitation and weeks for *Culex* spp. populations. The cross-correlation reads as: X [of the country site (in the row) at time t + lag] correlates with X [of the country site (in the column at the bottom row) at time t] with X = Temperature, Precipitation, *Culex* population

In all study sites, ground weather stations and vector surveillance systems using mosquito trapping devices have been established: CDC light traps baited with CO_2_ (Italy: 23 fixed locations; France: 3 fixed locations; and Greece: 15 fixed locations), and CO_2_ baited house-made traps with no light (Serbia: 35 fixed locations). Here we present the weekly averages of *Cx. pipiens* activity across all years of trap deployment (Italy 2009–2014, Fig. 1b; France 2011–2014, Fig. 2b; Serbia 2000–2007, Fig. 3b–d; Greece 2011–2014, Fig. 4b, c) in relation to temperature and precipitation (monthly averages) from May to October. In order to better observe and quantify the similarities between the different study sites, in relation to mosquito population dynamics and weather profiles, lagged cross-correlation analysis was performed among the different data series (annual average of *Cx. pipiens* weekly activity, annual average of monthly temperature and precipitation) (Fig. 5).

In relation to temperature, all study sites seem to follow very similar temporal patterns with the monthly mean temperature peaking in mid-July on week 30 (maximum mean temperature: Italy 24.6 °C; France 23.16 °C; Serbia 21.93 °C; Greece 27 °C) and dropping significantly in late September-early October. There is a larger variability observed in precipitation patterns and intensity. Overall precipitation is highest in late spring (May) and early fall (September-October) and decreases during mid-summer, with the only exception of Serbia where rainfall peaks in the end of June. These observations were also supported by the cross-correlation analysis that showed an almost identical temperature pattern followed in all countries (CC = 0.86–0.98 at lag 0). No solid conclusions can be reached based on cross-correlation of precipitation time series due to the high variability observed in our data probably resulting from the focal nature of rainfall.

In Italy *Cx. pipiens* started increasing in late May on week 22 and then gradually peaked by the end of June on week 26 (the average captures for 6 years is 700 *Culex*/trap/night). A significant drop of the mosquito population sizes was observed in late July-early August, but it was in late September-early October that populations started to diminish. In the southern natural wetlands of Camargue the first major peak of the population size appeared in mid-June on week 25 (4 year average: 450 *Culex*/trap/night). The populations persisted in relatively high numbers with low fluctuations until late September. In the urban and semi-urban environments of Novi Sad, *Culex* population size followed a trend similar to the one observed in Italy: populations started increasing in the beginning of June on week 23, showed a distinct peak on week 27 (urban) and week 28 (semi urban) (8 year average: 146 and 241 *Culex*/trap/night for the urban and semi-urban environment, respectively) and dropped significantly in mid-August. A slightly different pattern was observed in the rural *Culex* populations of Novi Sad, where mosquito populations had two distinct major peaks, the first on week 26 (8 year average: 297 *Culex*/trap/night) and the second in late August on week 35 (8 year average: 252 *Culex*/trap/night). A similar pattern to Novi Sad rural was observed in the agricultural (rice) environment of Thessaloniki, with the first peak appearing on week 26 (4 year average: 4189 *Culex*/trap/night), and the second peak, more dominant, appearing on week 34 (4 year average: 8946 *Culex*/trap/night). When considering the output of the cross-correlation analysis it seems that there is a strong correlation in the population patterns observed with a small lag. For example the pattern observed in Greece seems to appear 1 week, 3 weeks and 4 weeks later, respectively in Serbia, Italy and France (Greece-Serbia CC = 0.57 with 1 week lag, Greece-Italy CC = 0.60 with 3 week lag, Greece-France CC = 0.69 with 4 week lag).

## Conclusions

From the descriptive and comparative analysis conducted in this paper across four European countries with recent outbreaks of WNV infection, we observed the following: (i) with the exception of Italy, where synchronous transmission of WNV lineage 1 and 2 occur, the three other countries face outbreaks associated with only one of the two lineages; (ii) the largest recent outbreaks were caused by lineage 2 in Serbia and Greece; (iii) *Cx. pipiens* is considered the most important vector during epidemics; (iv) *Cx. pipiens* is ubiquitous with prolific mosquito breeding sites in urban, natural, and rural/agricultural environments; (v) *Cx. pipiens* annual average dynamics and weather profiles are very comparable across the four countries; and (vi) a variety of vector control strategies are implemented in each country targeting both nuisance and pathogen-transmitting mosquitoes with the majority of the adulticiding interventions applied as emergency response measures as soon as cases are reported in humans.

The temporal and spatial distribution of mosquito populations is shaped by a variety of environmental factors such as the availability, type and productivity of breeding sites, the climate and weather conditions together with anthropogenic factors such as the control methods including the management of breeding sites. The intensity of WNV transmission in nature is even more complex as it dependents not only on the mosquito population density but also on several other factors including the mosquitoes behavior, the temporal and spatial distribution of the virus amplification hosts (birds) and their immunological status, and the availability of alternative sources of blood meals for the mosquitoes. As a result of the complex interactions between the driving factors we observe a large variability in the WNV circulation intensity in successive years and across different regions. This large variability is partly due to the fact that only a part of the WNV transmission is detected and this part fluctuates between countries and even at the subnational level. And that part of the transmission is limited for WNV due to the fact that the amplifying cycle of WNV involves mosquitoes and birds, essentially wild birds either migratory or resident. In these conditions, planning of effective vector control strategies can be very challenging.

Timing of vector control applications is an important cornerstone for the implementation of effective WNV control and emergency measures can be largely ineffective if delayed until the index case appears [34]. There is a need to refine our understanding of the most effective vector control tools in order to optimize our resources and design proactive, evidence based WNV control strategies.

To elucidate the impact of vector control on WNV transmission intensity it is important to primarily show its impact on vector population dynamics. The environments described in this paper are appropriate study sites of WNV ecology and vector population because (i) they have key required factors in common: intense circulation of WNV has been detected in the most recent years, a significant number of cases have been reported in both humans and animals, high levels of *Culex* mosquito activity have been recorded and similar temporal distribution patterns of the mosquito populations have been observed, and (ii) they differ on factors that can be compared between the sites: different breeding sites are present and a variety of vector control practices have been implemented in the different sites. Through this paper we provided with a broad qualitative characterization of these environments and showcased the similarities on the average annual pattern of weather and vector populations across the four different countries. An advanced and more detailed analysis of the data obtained from studying these environments considering also the inter-annual variations of weather and vector populations will lead to designing and validating empirical and mathematical models of mosquito population dynamics. These models, after validation through field trials, will be made available for the public health professionals in Europe as a support tool to compare and assess the cost-effectiveness of different control strategies against WNV in Europe. Complementary beneficiaries of this project are researchers and others that will have access to a practical tool validated in the field in collaboration with a set of European countries.

## Abbreviations

*B.t.i.*: *Bacillus thuringiensis israelensis*
CC: Cross-correlation
ULV: Ultra low volume
VeCA: Vector control analysis
WNV: West Nile virus
